# Fabrication, Structure and Mechanical and Ultrasonic Properties of Medical Ti6Al4V Alloys Part I: Microstructure and Mechanical Properties of Ti6Al4V Alloys Suitable for Ultrasonic Scalpel

**DOI:** 10.3390/ma13020478

**Published:** 2020-01-19

**Authors:** Zheyu He, Hao He, Jia Lou, Yimin Li, Dongyang Li, Yongzhi Chen, Shaojun Liu

**Affiliations:** 1 State Key Laboratory for Powder Metallurgy, Central South University, Changsha 410083, China; holmes416@csu.edu.cn (Z.H.); liyimin333@163.com (Y.L.); dongyangl@csu.edu.cn (D.L.); csuchen@csu.edu.cn (Y.C.); 2 Research Center for Materials Science and Engineering, Guangxi University of Science and Technology, Liuzhou 545006, China; 3 School of Materials Science and Engineering, Xiangtan University, Xiangtan 411105, China; lou3166@xtu.edu.cn

**Keywords:** medical Ti6Al4V alloys, ultrasonic scalpel, heat treatment, ultrasonic properties, tensile strength, elastic modulus

## Abstract

Ti6Al4V alloy has been considered as a key component used in ultrasonic scalpels. In this series of papers, the fabrication, structure, and mechanical and ultrasonic properties of medical Ti6Al4V alloys suitable for ultrasonic scalpel are studied systemically. These alloys with low elastic modulus and present a typical bimodal microstructure with relatively high β phase content (~40%) and lamellar α thickness of ≤ 0.9 µm. In the first paper, the relationship between the microstructure and mechanical properties of hot-rolled Ti6Al4V alloys treated by heating treatment is discussed. In the second paper, the dependence of the ultrasonic properties on the microstructure of the heat-treated Ti6Al4V alloys is reported. With increasing solid solution temperature, the content and size of the primary α phase decrease. In contrast, the content and size of the lamellar α phase increase. Additionally, the β phase content first increases and then decreases. The microstructure of Ti6Al4V alloys could be slightly changed by aging treatment. When the solid solution treatment temperature increases to 980 °C from 960 °C, the average size of the lamellar α phase in the alloys increases by 1.1 µm. This results in a decrease in the average yield strength (93 MPa). The elastic modulus of alloys is mainly controlled by the β phase content. The microstructure of alloys by solution-treatment at 960 °C shows the highest β phase content and lowest average elastic modulus of 99.69 GPa, resulting in the minimum resonant frequency (55.06 kHz) and the highest average amplitude (21.48 µm) of the alloys at the length of 41.25 mm.

## 1. Introduction

Since the 1990s, ultrasound surgery operation has become widely used in the field of biomedicine. The development of an efficient, precise, and flexible ultrasonic scalpel (UAS) has become a hot topic ever since [1]. The ultrasonic scalpel system consists of three parts: the ultrasonic generator, transducer, and scalpel. Ultrasonic vibrations generated by the transducer excite a resonant horn and provide the required oscillatory displacement at the tuned ultrasonic frequency in the scalpels [2]. The scalpel is a direct-acting workpiece, which requires it present not only good biocompatibility and mechanical properties, but also high ultrasonic energy utilization and large amplitude displacement [3]. Annealed Ti6Al4V is commonly used for ultrasonic scalpel due to its high elastic strain limit, low acoustic attenuation, and biocompatibility [4]. 

Recent studies [4,5,6,7] show that there is a close relationship between the ultrasonic parameters and the microstructure of titanium alloys. Wilkie et al. [4] fabricated five distinct kinds of the microstructure of Ti6Al4V alloys ultrasonic scalpel through heat treatment. They further concluded that the samples with the equiaxial structure show better acoustic properties and higher acoustic attenuation than the alloys with fully lamellar samples. Lobkis et al. [5] reported that the ultrasonic properties of the Ti-based alloys show a strong frequency-dependence of backscattering on the microstructure. Hector et al. [6] showed that the ultrasonic attenuation was different between Widmanstätten and equiaxed microstructures of Ti6Al4V alloy, which was mainly attributed to the scattering loss due to the precipitation of Ti_3_Al particles homogeneously distributed in the α phase. Bhattacharjee et al. [7] studied the ultrasonic attenuation of near-α titanium alloy and found that medical Ti alloys with larger micro-textured microstructure present higher attenuation than those with smaller micro-textured microstructure. 

It is well known that the microstructure of titanium alloys can be influenced significantly by the composition, processing, and heating treatment [8,9,10]. For Ti6Al4V alloys, various microstructures can be obtained by different heat treatments based on the β-transus temperature [11,12,13]. Matsumoto et al. [14,15,16,17] fabricated ultrafine-grained structure (UFG) of Ti6Al4V alloys through different heat treatment at 800 °C. Although the UFG alloys exhibit low-temperature superplasticity, the plasticity at room temperature is still very poor (~1% elongation). Ren et al. [18] obtained a bimodal microstructure of Ti6Al4V alloys by annealing below the β-transition point. Further investigation showed that the plasticity and strength of alloys are closely related to the content of the primary α phase. In contrast, Peng et al. [19] reported on the fabrication of the Widmannstatten structure of Ti6Al4V-DT alloys by annealing in the β phase region. It was found that the strength and plasticity of alloys decrease with increasing test temperature. Obviously, the heat treatment is an effective way to tailor the microstructure of the alloys. 

Additionally, as for two-phase titanium alloys, factors that influence the mechanical properties include the volume fraction of transformed β phase, size of prior β grain, primary α phase, secondary α phase, etc. [20,21]. However, conclusions made by different researchers often are inconsistent. Peng et al. [22] found that the strength of Ti6Al4V alloys is related to the primary α phase content in the alloys. In contrast, Guo et al. [23] concluded that the tensile strength of alloys has very low dependence on the primary α phase content. However, the dependence of the tensile ductility on the primary α content is strong. In contrast, Vrancken et al. [24] showed that the key factor affecting the strength of Ti6Al4V alloys is the size of α and β phases. It is found that the σ_y_ and UTS of Ti6Al4V alloys decrease while the fine needle-like α phase changes into a coarser mixed structure of α + β phases. Niinomi et al. [25] found that the strength of Ti6Al4V alloys increases as the size of β grain increases. 

The ultrasonic properties of medical Ti alloys can be significantly improved by adjusting the microstructure of Ti alloys. However, very little attention has been paid to the influence of the fabrication processing and structure on the ultrasound properties of the Ti6Al4V alloys. There is still a lack of systematic studies on the relationship between the phase content, size, and mechanical and ultrasonic properties of Ti6Al4V alloy. These two papers aim to prepare Ti6Al4V alloys with significantly different microstructure combining the solid solution with aging treatment. Special attention is paid to the clarification of the mechanisms that are helpful for developing low-cost and highly efficient flexible ultrasonic scalpel (FUS) tools. In the first paper, special attention is paid to the relationship between the microstructure and mechanical properties of the Ti6Al4V alloys. In the second paper, further study will focus on the influence of microstructure on ultrasonic properties. 

## 2. Experiments

**Figure 1:**
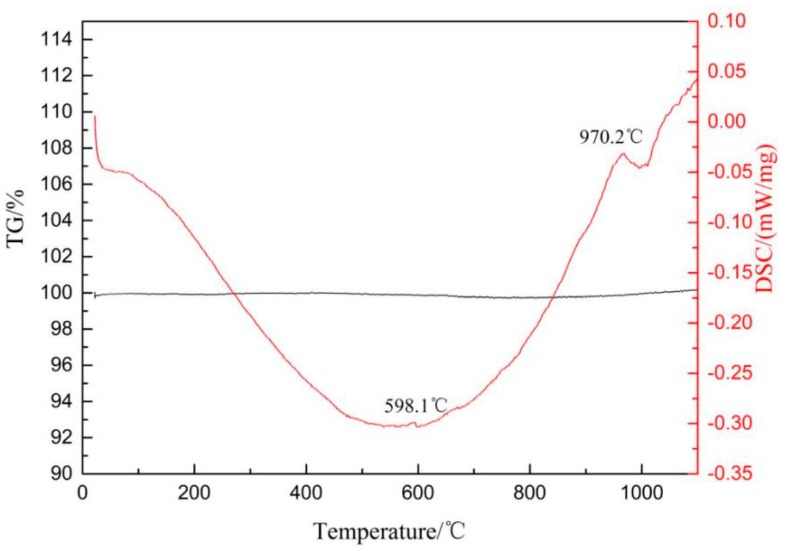
DSC-TG graph of as-received Ti6Al4V alloy.

**Figure 2:**
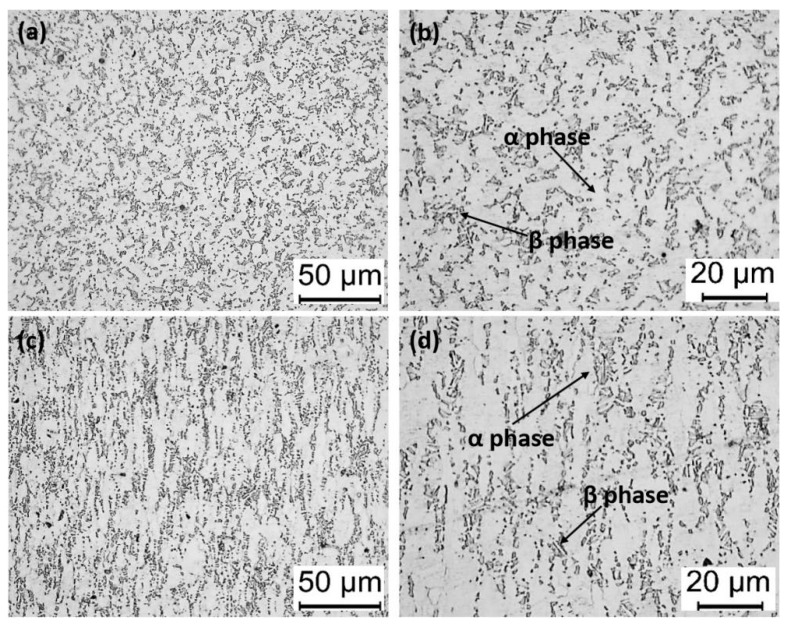
Microstructure of as-received Ti6Al4V alloy (**a**,**b**) viewed from the radial direction and (**c**,**d**) viewed from the axial direction.

Table 1 lists the chemical composition of the Ti6Al4V alloys in the present investigation. As shown in Figure 1, the β and α phase transus temperature of the Ti6Al4V alloys are 970.2 °C and 598.1 °C, respectively. Cylinder samples (Ø8.5 × 60 mm) cut from the as-received round bar and their microstructure are shown in Figure 2. In general, the microstructure of Ti6Al4V alloys contains a large number of the α phase and a small number of the β phase evenly distributed between the α phases. However, the microstructure along the axial and radial direction is quite different. As shown in Figure 2a,b, when it is viewed from the radial direction, a large number of the equiaxial α grain with particle size ~5–10 µm can be observed, and the α phase is interspersed with the transformed β phase. In contrast, when it is viewed from the axial direction, the microstructure has an elongated α phase and intergranular transformed β phase, as shown in Figure 2c,d, respectively. The difference between the radial and axial directions of the microstructure of as-received specimens is due to the hot-rolling process. However, since the ultrasonic scalpel longitudinal vibration along the axial in the clinics, this study mainly focuses on the microstructure and properties of the Ti6Al4V alloys along the axial direction.

Heat treatment typically includes solution treatment and aging treatment, as shown in Table 2. The solution treatment is in a range from 920 °C to 980 °C for 1 h, followed by air cooling (AC, the cooling rate at about 0.5 °C/s) to achieve different microstructures. The solid solution specimen treated at 960 °C was further subjected to additional aging treatment for 2 h in a range from 600 °C to 750 °C, and then air cooling. Fifteen samples were used for each heat treatment condition for the subsequent tests. The as-received and heat-treated specimens were ground with SiC papers and then polished with a SiO_2_ pyrolysis suspension and etched with reagent 10% HF + 40% HNO_3_ + 50% H_2_O solution for 5–10 s at room temperature. The microstructure of Ti6Al4V alloys was observed by Leica DM2700M optical microscope (Leica Microystems, Weztlar, Germany) and further analyzed by VEGA3 LMH/LMU scanning electron microscope (TESCAN ORSAY HOLDING, a.s., Brno, Czech Republic). 

The macro and micro texture of the α phase was measured by the D8 Bruker XRD system (Bruker AXS, Oestliche, Germany) and NordlysNano SEM (Oxford Instruments plc, Abingdon, UK) with an Oxford EBSD (Electron Backscattered Scattering Detection) detector, respectively. The specimens for EBSD were prepared by mechanically grounding and polishing, and then electro-polished in a solution of 6% perchloric acid, 34% butarol, and 60% carbinol at 45 V and −40 °C for 15 s. The EBSD testing angle is 70°, the accelerating voltage and acquisition speed of the test are 20 kV and 11.27 Hz, respectively. The EBSD orientation maps, pole figure and inverse pole figure maps were analyzed by HKL Channel 5 software (Channel 5 11 b-win7 32). The α and β content and their size of each in Ti6Al4V alloys were further analyzed by Image J (v 1.8.0) software according to the GB/T 6394-2017 specification [26].

Samples were cut from the as-received and heat-treated specimens along the rolling direction and machined into the tensile specimens. Then subjected to tensile tests using the WDW-100G computerized Instron testing system with the maximum testing force of 100 kN at a strain rate of 0.01 s^−1^ according to the GB/T 228.1-2010 specification [27]. The results of tensile tests were taken from 3 tensile specimens in order to guarantee the reliability of experimental results. The elastic modulus was determined by DTM-II Dynamic Elastic Modulus Tester (Xiangtan Instrument Co., Ltd., Xiangtan, China) according to the GB/T 22315-2008 specification [28]. The tensile strength, elastic modulus, and elongation of the as-received Ti6Al4V alloys are 986.42 MPa, 110.18 GPa, and 15.5%, respectively.

## 3. Results and Discussion

### 3.1. Microstructure and Mechanical Properties after Solution Treatment

**Figure 3:**
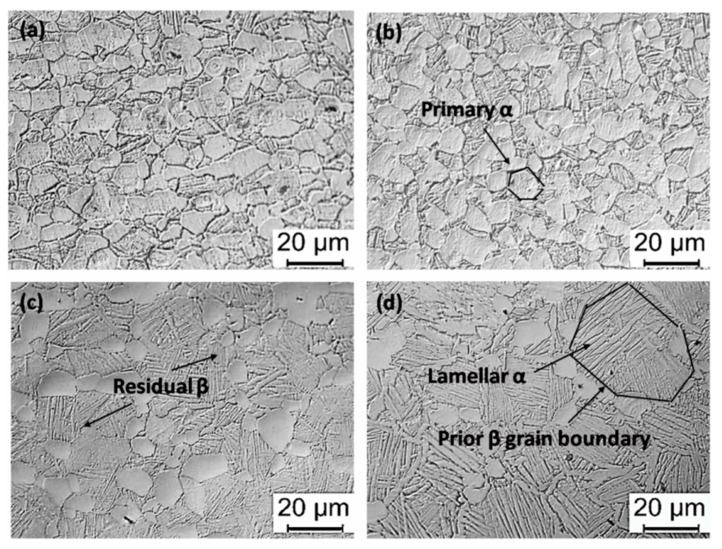
Metallographic image of microstructure obtained by different solution treatments (**a**) 920 °C × 1 h, AC; (**b**) 940 °C × 1 h, AC; (**c**) 960 °C × 1 h, AC; and (**d**) 980 °C × 1 h, AC.

Figure 3 shows the microstructure of the Ti6Al4V alloys along the axial direction after different solution treatments. After solid-solution treatment, the microstructure contains primary α, lamellar α, and transformed β phase. It is observed that the primary α phase in the Ti6Al4V alloys treated by solid solution treatment at 920 °C loses its orientation and evolves into an equiaxial shape. When the solid solution treatment temperature increases, the microstructure of as-treated Ti6Al4V alloys gradually transforms from equiaxial to bimodal. The microstructure of the as-treated specimen at 980 °C that is above the β transus temperature presents the Widmannstatten structure characteristics, as shown in Figure 3d. This is consistent with the reported solid solution treatment parameters obtaining two-phase Ti6Al4V alloys with different microstructures [12].

**Figure 4:**
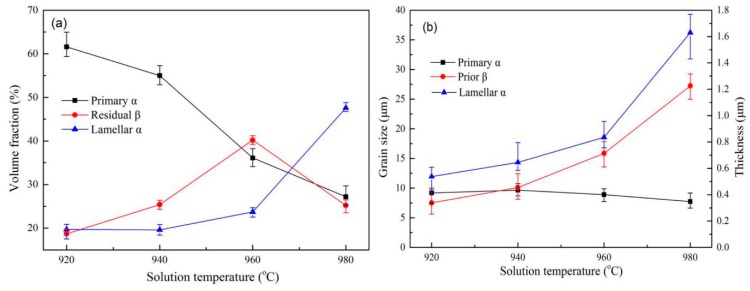
Microstructure statistics (**a**) volume fraction of α, β phase under different solution temperature and (**b**) grain size and thickness of α, β phase under different solution temperature.

The phase composition and grain size of treated Ti6Al4V alloys are shown in Figure 4. As the solid solution treatment temperature increases, the content and grain size of the primary α phase in the alloys decrease. However, a reverse trend is observed for the lamellar α phase in the treated alloys where the prior β grain grows and the β phase content first rises and then falls. A maximum value of 40.2% is observed when the alloys are treated by solid solution treatment at 960 °C. It was reported [29] that the higher the solid solution treatment temperature, the lower the equiaxial α content and the larger the lamellar α thickness. These observations are consistent with the present investigation. It is well known that the Ti6Al4V alloys exhibit an α phase (hcp) at low temperature, which can transform into a β phase (bcc) at elevated temperature [20]. The phase transformation during heating (α~β) and cooling (β~α) is governed by the so-called Burgers orientation relationship {0 0 0 2}_α_ || {1 1 0}_β_ and <1 1 -2 0>_α_ || <1 1 1>_β_ [29]. There are 12 possible α orientations that can transform from a single parent β grain during β~α phase transformation while cooling so that the microstructure of alloys exhibit different shapes of α phase (equiaxial, lamellar et al.) and residual β phase after solid solution treatment. During heating below the β transus (970.2 °C in this article), the higher the solution temperature is, the more α phase is transformed into β phase. This makes the residual β phase reach the maximum value of about 40% in 960 °C solid solution treated specimen by air cooling. However, when the solid solution temperature exceeds the β transus temperature, the α phase completely transforms into the β phase. There is sufficient time for metastable β phase transformed into lamellar α during air cooling, resulting in a decrease in the residual β phase content and the further increase in the thickness of lamellar α. Therefore, as the solution temperature rises from 960 °C to 980 °C, the β phase content decreases from 40.2% to 25.2%, while the content and thickness of lamellar α phase increase to 47.6% from 23.7% and to 1.63µm from 0.836 µm, respectively.

**Figure 5:**
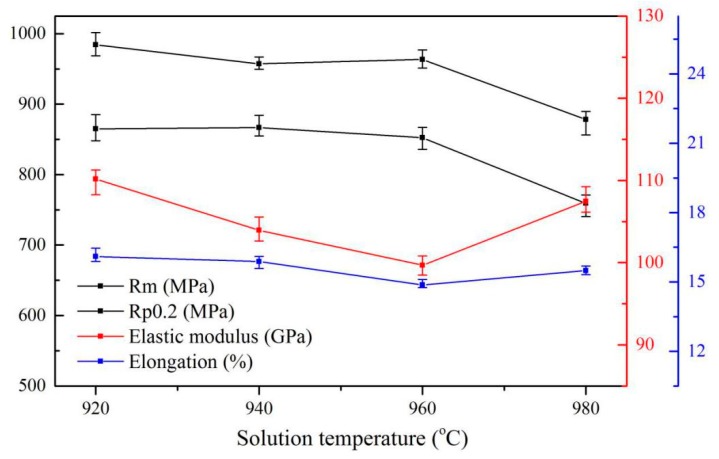
Mechanical properties of microstructures obtained by different solution treatments.

Figure 5 shows the dependence of the mechanical properties of Ti6Al4V alloys treated by solid solution treatment on the microstructures that have been shown in Figure 3. It was observed that the tensile strength of the alloys treated below the β transus temperature (920–960 °C) is significantly higher than those treated in temperature above 980 °C. The alloys by solid solution treatment at 960 °C have the lowest average elastic modulus (99.69 GPa). In contrast, the plasticity of the alloys does not show strong microstructure dependence. As expected, these results clearly show that the content and size of the α and β phases can significantly affect the mechanical properties of the alloys with different solid solution treatment temperature. Additionally, the curve in Figure 4 further implies that the lamellar α phase rather than the primary α phase and residual β phase is the main factor affecting the tensile strength of the alloys. The primary α phase seems to have little effect on the strength of the alloy. This agrees with the reported results [18]. Niinomi et al. [25] proposed that the yield strength of β type alloys increased with increasing prior β grain size and attributed the increase of the yield strength to the effect of precipitated α rather than β grain size. However, the results in the present study show that the strength of the alloys decreases with the mobility of the prior β phase grain boundaries, which is consistent with the reported results [19]. It is known that under the air-cooling condition, the growth of β grains accompanies a full growth of the lamellar α phase in the Ti6Al4V alloys. As the thickness of the lamellar α phase increases, the α/β phase boundaries and the dislocation slip resistance decreases [19]. This subsequently makes the dislocations difficult to pile up and decrease the strength of as-treated alloys. Similar mechanisms have been proposed by Donachie et al. [20] that the strength of titanium alloys has a strong dependence on the number and fineness of the α/β phase boundaries. 

**Figure 6:**
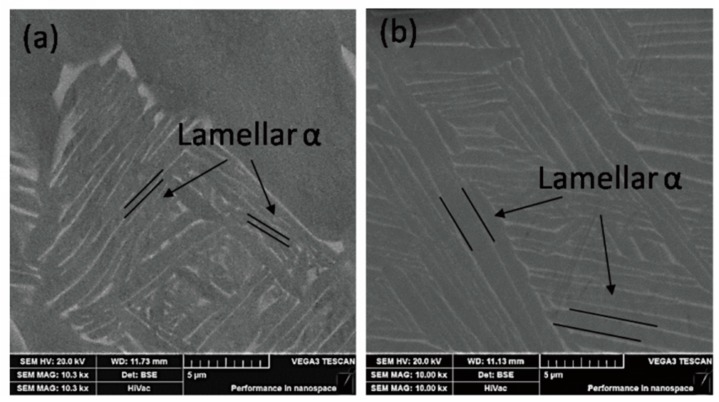
BSE image of microstructures obtained by different solid solution treatment: (**a**) 940 °C ×1 h, AC; and (**b**) 980 °C × 1 h, AC.

Figure 6a,b are the BSE (Back scattered Electron Imaging) images of the microstructures of the Ti6Al4V alloys treated by solid solution treatment at 940 °C and 980 °C, respectively. The bright and dark regions denote the β phase and α phase, respectively. As shown in Figure 6a,b, it is obvious that the thickness of the lamellar α phase in the Ti6Al4V alloys increases significantly after the solution treatment at 980 °C. The average thickness of the lamellar α phase increases by 1.09 µm for the alloys treated at 980 °C comparing with the alloys treated at 920 °C. It can be determined that the average tensile strength of the specimen solution-treated at 920 °C decreases significantly from 984.11 MPa to 878.15 MPa of the specimen solution-treated at 980 °C. It is ascribed to the large increase in the content and thickness of lamellar α, roughly 140% and 202% respectively. However, the elongation of the alloys just changes slightly and remains about 15%, a value that is consistent with the reported [19] ones (13.25%~17.75%). In contrast, the elastic modulus of the alloys first decreases, and it subsequently increases with increasing solution temperature, a trend that is consistent with that of the residual β phases shown in Figure 4a. Several reports [30,31,32] have shown that the elastic modulus of the β phases in the titanium alloys is nearly 80 GPa, which is lower than that of the α phase (~120 GPa). Therefore, the increase of the β phase content in titanium alloys can reduce the elastic modulus of the titanium alloys. After solution treatment at 960 °C, the average elastic modulus of the alloys is 99.69 GPa, lower than other solid solution treated specimens, which results from the maximum β phase content (40.2%) in the Ti6Al4V alloys.

It is noticed that the microstructure of the as-received Ti6Al4V alloys (Figure 2) and their counterparts treated by solid solution treatment at 920 °C (Figure 3a) presents obvious differences. For example, the shape of primary α phases in these two alloys are elongated and equiaxed; the β phase content in these alloys is 8.38% and 15.1% respectively, which is significantly different as well. However, the elastic modulus of the alloys with significant different microstructure is almost the same (~110 GPa), which does not decrease significantly with the increase of the β phase content in the alloys. It is possible that the orientation of the α phase could play an important role in affecting the elastic modulus of the Ti6Al4V alloys. It was reported [31] that the α phase has strong elastic anisotropy in two-phase titanium alloys. Therefore, it is believed that both the orientation of the α phase and β phase content could determine the elastic modulus of the Ti6Al4V alloys.

**Figure 7:**
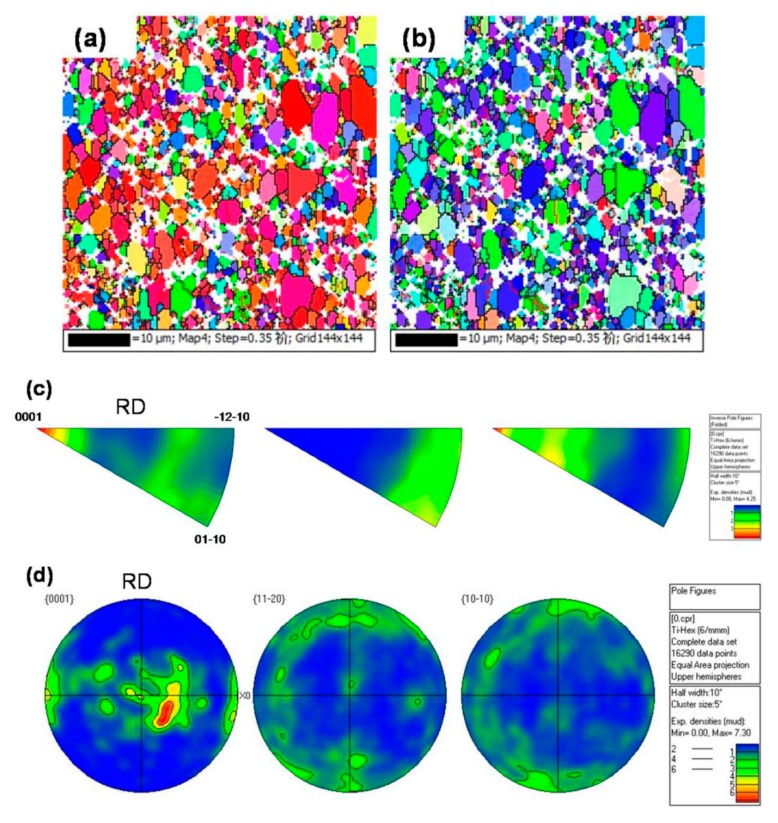
The EBSD results showing the microstructure and texture distribution of the α phase of the as-received alloys: (**a**,**b**) inverse pole figure (IPF) map from RD and TD direction; (**c**) inverse pole figure (IPF) sheet and (**d**) pole figure (PF).

**Figure 8:**
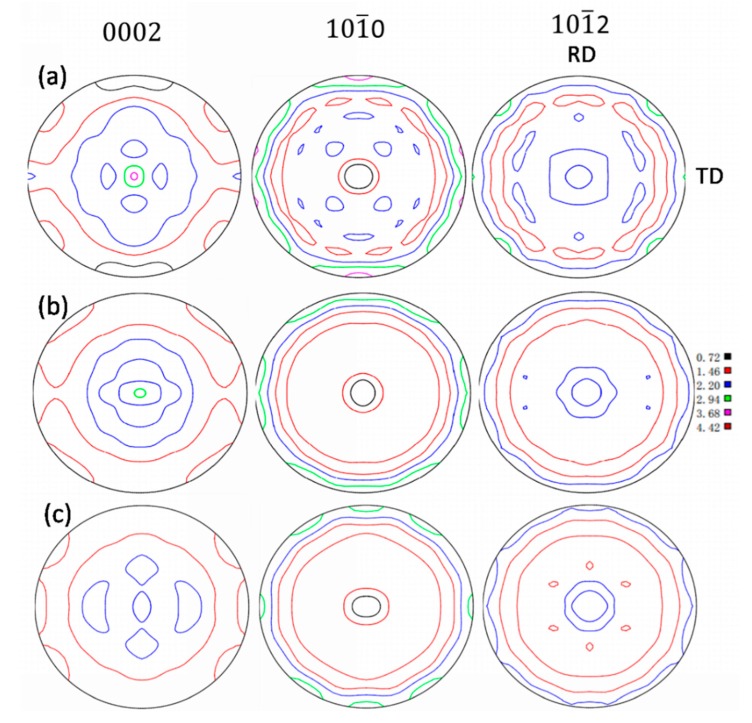
Pole diagram of different microstructure: (**a**) as-received Ti6Al4V alloy; (**b**) 920 °C × 1 h, AC solid solution treatment; and (**c**) 940 °C ×1 h, AC solid solution treatment.

To further analyze the orientation of the α phase, the texture of the α phase in the as-received and the solution-treated Ti6Al4V alloys are observed by EBSD and X-ray diffraction analysis, respectively, as shown in Figure 7 and Figure 8. Figure 7a–d show the morphology of the rolling plane, and the IPF sheet and pole figure of as-received alloys respectively. It is obvious that there are {0 0 0 1} textures of α phases in the as-received alloys as shown in Figure 7c,d. This observation is consistent with the calculation pole figure as shown in Figure 8a. However, the textures of α phases in the alloys gradually disappear, after solid solution treatment at a temperature above 920 °C, as shown in Figure 8b,c. These results are consistent with those shown in Figure 2 and Figure 3 in which the primary α phases of as-received alloys transformed into equiaxed shapes from elongated shapes after solid solution treatment.

It has been pointed out [30] that the *c*:*a* of the HCP titanium (α phase) is 1.59, which is less than the ideal value (1.63) because the *a* is longer, resulting into the atomic distance in the basal plane (0001) increase and the interatomic forces decrease, respectively. The elastic modulus of this plane therefore decreased. The {0 0 0 1}_α_ textures appear in the as-received alloys, which means that the {0001}_α_ (showing lower elastic modulus) parallel to the rolling plane (test direction) is beneficial to reduce the elastic modulus of the Ti6Al4V alloys. 

As shown in Figure 8a,b, the α phase textures of the as-received Ti6Al4V alloys disappear after solid solution treated at 920 °C, which might lead to an increase in the elastic modulus of the alloys. However, the residual β phase content gradually increases with the solid solution temperature increased to 920 °C and cause a decrease of elastic modulus of the alloys. These two factors together lead to the elastic modulus of as-received alloys and the alloys solid solution treated at 920 °C is basically the same.

### 3.2. Microstructure and its Mechanical Properties after Aging Treatment

**Figure 9:**
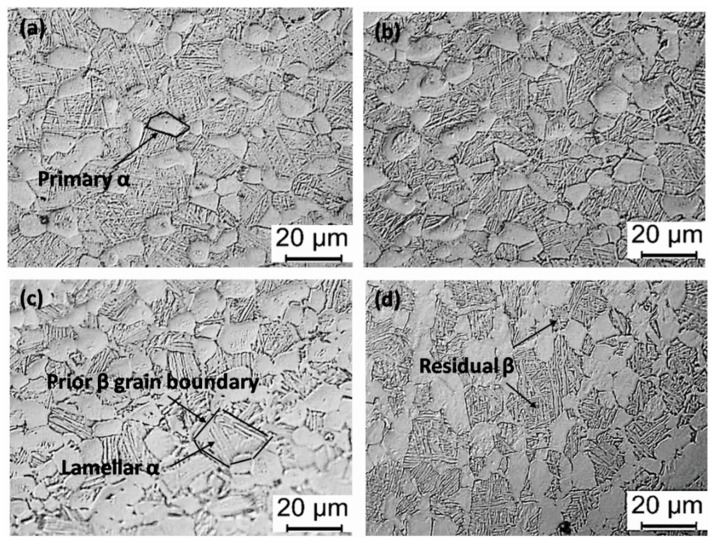
Microstructure under the different solution treatments followed by aging. (**a**) 960 °C × 1 h, AC + 600 °C × 2 h, AC; (**b**) 960 °C × 1 h, AC + 650 °C × 2 h, AC; (**c**) 960 °C × 1 h, AC + 700 °C × 2 h, AC; and (**d**) 960 °C × 1 h, AC + 750 °C × 2 h, AC.

**Figure 10:**
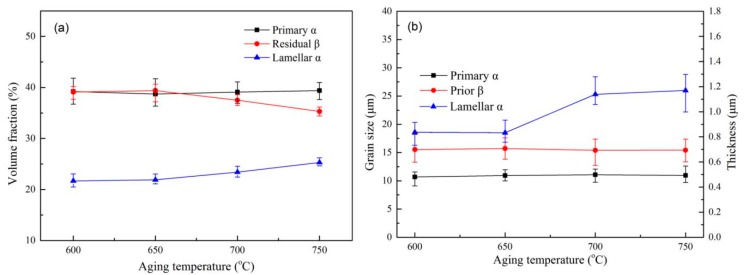
Microstructure statistics (**a**) volume fraction of α, β phase under different aging temperature and (**b**) grain size and thickness of α, β phase under different aging temperature.

To further analyze the influence of the α and β phase content and their size on the mechanical properties of the Ti6Al4V alloys, the specimen subjected to solution treatment at 960 °C was further treated by aging treatment. The microstructure of the alloys after aging treatment consists of primary α phase, lamellar α phase, and residual β phase, as shown in Figure 9. The microstructure of the Ti6Al4V alloys still retains the bimodal characteristics after aging at different temperatures, with no significant difference in morphology. After aging treatment, the content of the primary α phase (~39%) increases slightly compared with the alloys (~36%) treated by the solid solution treatment at 960 °C. At the same time, the average size of the primary α grain of the alloys after aging-treated at 600 °C increases to 1.78 µm compared with the specimen solution-treated at 960 °C. Statistical data on the content and size of each phase in the aging microstructure is shown in Figure 10. It is stressed that when the aging temperature increases to 750 °C from 600 °C, the volume fraction and size of the primary α phase remains unchanged at about 39% and 11 µm. These results show that in a certain aging temperature range, the volume fraction and size of the primary α phase in the Ti6Al4V alloys have no significant dependence on the aging temperature, a conclusion that is consistent with the observation that the volume fraction of the primary α is mainly affected by the solution temperature [18]. In contrast, when the aging temperature increases to 750 °C from 600 °C, the thickness of the lamellar α phase increases and the content of the β phase decreases slightly. The average thickness of the lamellar α phase increases to 1.17 µm from 0.836 µm. It is possible that the enhanced aging temperature is favorable to the phase transformation of the metastable β phase into the lamellar α phase.

**Figure 11:**
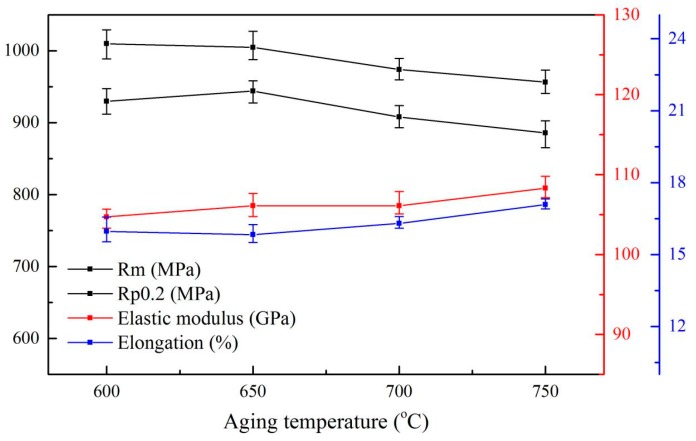
Mechanical properties of microstructures obtained by different aging treatments.

**Figure 12:**
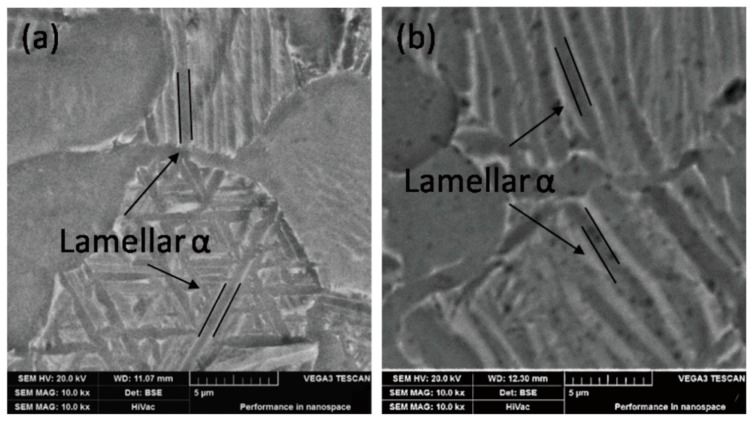
BSE image of microstructure under the different solution treatments followed by aging (**a**) 960 °C × 1 h, AC + 600 °C × 2 h, AC; (**b**) 960 °C × 1 h, AC + 750 °C × 2 h, AC.

After aging treatment, the plasticity of the specimen does not change much compared with the alloys treated by the solid solution treatment at 960 °C, and the elongation remains at about 16%, while the tensile strength and the elastic modulus of the alloys increase. One possible reason is that the metastable β decomposed into the α phase during the aging process, which can enhance the tensile strength of the alloys. The decrease in residual β leads to an increase in the elastic modulus of the alloys. The mechanical properties of the alloys after aging treatment are shown in Figure 11. The results show that the tensile strength of the specimens slightly decreased from 1009.72 MPa to 956.3 MPa with the increase in aging temperature (from 600 °C to 750 °C), which may be attributed to the increase in the content and thickness of lamellar α, roughly 17% and 40%, respectively. The growth of lamellar α resulting in a slight decrease in the α/β phase boundary, hence, the strength of the specimen after aging treatment was not significantly reduced. As shown in Figure 12, the trend of growth of the lamellar α phase with increasing aging temperature further shows that the lamellar α is the main factor affecting the strength of the Ti6Al4V alloy. Figure 11 also shows that as the aging temperature rose from 600 °C to 750 °C, the elongation can be maintained at 16.5 ± 0.7%. At the same time, the elastic modulus increased by about 3 GPa, which is attributed to the further decrease (~5%) of the residual β phase.

## 4. Conclusions

Given the strong dependence of ultrasonic properties on the microstructure of Ti6Al4V alloys used as an ultrasonic scalpel, in this paper, the effects of solid solution and aging treatment on the microstructure and mechanical properties of Ti6Al4V alloys were studied; the following conclusions can be drawn:

1. The content and size of the primary α phase of the Ti6Al4V alloy decrease with an increase in solid solution temperature. The lamellar α phase exhibited the opposite trend, i.e., the β phase content first increases and then decreases. The increase in the aging temperature has little effect on the content and size of the primary α phase but causes a slight increase in the thickness of the lamellar α phase.
2. For the solution and aging-treated Ti6Al4V alloys, the main factor affecting the tensile strength is lamellar α. As the solid solution temperature increases from 960 °C to 980 °C, the average thickness of lamellar α increased by 1.09 µm, and the average yield strength decreased by 93 MPa. As aging temperature increases from 600 °C to 750 °C, the thickness of lamellar α slightly increases by 0.33 µm and causes the yield strength of the specimens remained at about 900 MPa.
3. The elastic modulus of the Ti6Al4V alloy is mainly controlled by the texture of the α phase and content of the β phase. However, the β phase content is the main factor affecting the elastic modulus of the alloys treated by solution and aging treatment. Specifically, the specimen’s solid solution treated at 960 °C has the highest residual β content, hence the average elastic modulus is the lowest at 99.69 GPa. Additionally, the elastic modulus of the alloys after aging treatment remained at about 105 GPa, which is attributed to the stable residual β phase content.

## Figures and Tables

**Table 1:** Chemical composition of as-received Ti6Al4V alloy.

Ti	Al/%	V/%	Fe/%	C/%	N/%	H/%	O/%
Reminder	6.05	4.61	0.25	0.08	0.05	0.012	0.18

**Table 2:** Heat treatment scheme of various specimens.

Specimen No.	Heat Treatment
A	920 °C, 1 h, AC
B	940 °C, 1 h, AC
C	960 °C, 1 h, AC
D	980 °C, 1 h, AC
E	(960 °C, 1 h, AC) + (600 °C, 2 h, AC)
F	(960 °C, 1 h, AC) + (650 °C, 2 h, AC)
G	(960 °C, 1 h, AC) + (700 °C, 2 h, AC)
H	(960 °C, 1 h, AC) + (750 °C, 2 h, AC)
